# Parsing the Roles of the Transcription Factors GATA-4 and GATA-6 in the Adult Cardiac Hypertrophic Response

**DOI:** 10.1371/journal.pone.0084591

**Published:** 2013-12-31

**Authors:** Jop H. van Berlo, Bruce J. Aronow, Jeffery D. Molkentin

**Affiliations:** 1 From the Department of Pediatrics, Cincinnati Children’s Hospital Medical Center, University of Cincinnati, Ohio, United States of America; 2 Howard Hughes Medical Institute, Cincinnati Children’s Hospital Medical Center, Cincinnati, Ohio, United States of America; Loyola University Chicago, United States of America

## Abstract

The transcriptional code that programs cardiac hypertrophy involves the zinc finger-containing DNA binding factors GATA-4 and GATA-6, both of which are required to mount a hypertrophic response of the adult heart. Here we performed conditional gene deletion of *Gata4* or *Gata6* in the mouse heart in conjunction with reciprocal gene replacement using a transgene encoding either GATA-4 or GATA-6 in the heart as a means of parsing dosage effects of GATA-4 and GATA-6 versus unique functional roles. We determined that GATA-4 and GATA-6 play a redundant and dosage-sensitive role in programming the hypertrophic growth response of the heart following pressure overload stimulation. However, non-redundant functions were identified in allowing the heart to compensate and resist heart failure after pressure overload stimulation, as neither *Gata4* nor *Gata6* deletion was fully rescued by expression of the reciprocal transgene. For example, only *Gata4* heart-specific deletion blocked the neoangiogenic response to pressure overload stimulation. Gene expression profiling from hearts of these gene-deleted mice showed both overlapping and unique transcriptional codes, which is presented. These results indicate that GATA-4 and GATA-6 play a dosage-dependent and redundant role in programming cardiac hypertrophy, but that each has a more complex role in maintaining cardiac homeostasis and resistance to heart failure following injury that cannot be compensated by the other.

## Introduction

The adult heart typically undergoes a process of hypertrophic growth in response to injury or disease stimulation that promotes neurohumoral activation and/or increases in wall stress [1]. The heart responds to disease or stress by activating signaling and transcriptional pathways that ultimately result in the activation of pro-growth and adaptive genes in an attempt to compensate for an injury event or disruption of homeostasis [1]. The zinc-finger containing transcription factors GATA-4 and GATA-6 are each expressed in the heart where they play a prominent role in myocyte lineage commitment and differentiation during embryogenesis [2], [3] but each is also induced and re-employed in the adult heart following injury where they participate in mediating the hypertrophic growth of individual myocytes [3]. We have shown previously that heart-specific deletion of *Gata4* in the adult mouse renders the heart less able to hypertrophy with agonist or pressure overload stimulation, as well as more likely to succumb to heart failure, even with aging [4]. Loss of *Gata4* from the heart also negatively impacted neoangiogenesis following stress stimulation, further defining its role in homeostasis and prevention of heart failure [5]. By comparison, cardiac-specific deletion of *Gata6* similarly resulted in a defective hypertrophic response with pressure overload stimulation, as well as a greater propensity towards heart failure [6]. During development *Gata4* and *Gata6* seem to be completely redundant in programming cardiomyocyte commitment, as deletion of either gene alone still permitted myocyte formation, yet deletion of both together resulted in a complete loss of the lineage [7]. Moreover, *Gata4^+/−^ Gata6^+/−^* (double heterozygous) mice are embryonic lethal, similar to homozygous null mutations in either gene alone, again suggesting functional redundancy and that the total dosage of the four *Gata4/6* alleles is most critical with respect to their function [8]. Here we attempted to investigate if GATA-4 and GATA-6 had unique functionality in the adult heart during the hypertrophic response and/or in maintaining proper homeostatic gene expression that underlies cardiac “well-being” by using a reciprocal gene replacement strategy.

## Materials and Methods

### Animal Models and Methods

*Gata4-loxP* (fl) and *Gata6-loxP* (fl) mice were each described previously, as were transgenic mice expressing a tetracycline transactivator (tTA) driven inducible and cardiac-specific α-myosin heavy chain (αMHC) promoter or deletion with the βMHC-promoter driven, cre-expressing transgene [4], [6]. Pressure overload induced by transverse aortic constriction (TAC) was performed as described previously, as well as assessment of cardiac ventricular performance by echocardiography [4]–[6]. Assessment of capillary density in the heart with isolectin B4 on frozen histological sections was performed as described previously [5]. Western blotting from cardiac nuclear protein extracts was performed as described previously [9]. mRNA collection from adult hearts and subsequent Affymetrix mouse set ST1.0 chips microarrays were generated and analyzed as described previously [9]. qRT-PCR was performed for selected genes using SYBR green (Applied Biosystems). We used 6 control mice and 6 knock-outs (3 for each group) for the microarray experiments (2 βMHC-cre, 2 *Gata4-loxP*, 3 *Gata4-loxP* βMHC-cre, 2 *Gata6-loxP*, and 3 *Gata6-loxP* βMHC-cre). We also used at least 4 mice per genotype in the qRT-PCR experiments. The microarray data was deposited in the GEO database through the NCBI gateway under accession number GSE52317.

#### Ethics statement

All animal experimentation was approved by the Office of Research Compliance and Regulatory Affairs and by the Cincinnati Children’s Hospital Institutional Animal Care and Use Committee (Protocol Number: 2E11104). No human subjects were used.

#### Statistical tests

Statistical significance was determined by ANOVA and Newman-Keuls pairwise comparisons for multivariate experiments and t-test for experiments with 2 groups.

## Results

**Figure 1:**
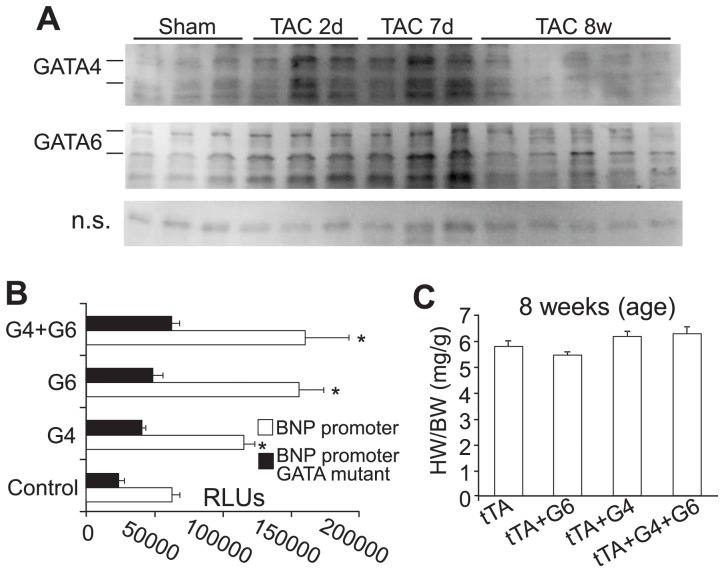
GATA-4 and GATA-6 are similarly induced in the adult heart with pressure overload. A, Western blot analysis of GATA-4 and GATA-6 protein expression in the adult mouse heart at baseline or after pressure overload stimulation for the indicated times. A non-specific (n.s.) band from the GATA-6 western was used to show equal loading and running conditions. B, Relative luciferase units (RLU) from a plasmid containing the luciferase reporter fused to the b-type natriuretic peptide (BNP) promoter or a mutant promoter lacking functional GATA binding sites, which was co-transfected into neonatal rat cardiomyocytes with expression plasmids encoding GATA-4 (G4), GATA-6 (G6), or both together. *P<0.05 versus empty vector transfected control. N = 3. C, Heart weight normalized to body weight (HW/BW) of the indicated genotype of cardiac-specific transgenic mice expressing either GATA4, GATA6, or both together at 8 weeks of age.

We have previously shown that both GATA-4 and GATA-6 proteins are upregulated in the adult heart during the cardiac hypertrophic response [4], [6]. Here we investigated the induction of GATA-4 and GATA-6 in the heart more carefully after pressure overload stimulation, which showed nearly identical profiles in the degree of protein upregulation as well as the time course of induction, which begins within the first 1–2 days, then is fulminant by day 7, thereafter trailing off by 8 weeks (Fig. 1A). GATA-4 and GATA-6 were previously shown to function in a synergistic manner as transcriptional inducers when co-overexpressed in cultured myocytes, suggesting that they are not functional redundant in that they work together more effectively than either one individually [10]. However, in our hands GATA-4 and GATA-6 each induced expression of the b-type natriuretic peptide (BNP) promoter in cultured cardiomyocytes equal to the combination of GATA-4 with GATA-6 (Fig. 1B). These same 2 GATA-4/6 expression plasmids previously produced approximately equal levels of promoter activity in neonatal cultured cardiomyocytes when transfected with the BNP promoter or an artificial GATA-multisite reporter plasmid [11]. Similarly, if GATA-4 and GATA-6 did indeed have a functional synergy when co-overexpressed we reasoned that it might induce a hastened hypertrophic response *in vivo*. While both GATA-4 and GATA-6 overexpression in the heart, as driven by the αMHC promoter, can lead to mild cardiac hypertrophy by 6–8 months of age [4], [6], [11], mice containing both the GATA-4 and GATA-6 transgene did not show induction of hypertrophy at 8 weeks of age (Fig. 1C, and data not shown). Longer time points were not investigated because each transgene alone begins to show pathologic-like cardiac hypertrophy at 4–6 months of age. Indeed, if synergy was occurring between these 2 factors, 8 weeks of age should have produced a demonstrable effect, as many other hypertrophy inducing transgenes show fulminant hypertrophy as early as 1–2 weeks of age. Thus, in the adult heart GATA-4 and GATA-6 are each induced in a similar manner during stress-induced hypertrophy, but they do not appear to function together in a synergistic manner in driving cardiac hypertrophy more than either individual transgene.

**Figure 2:**
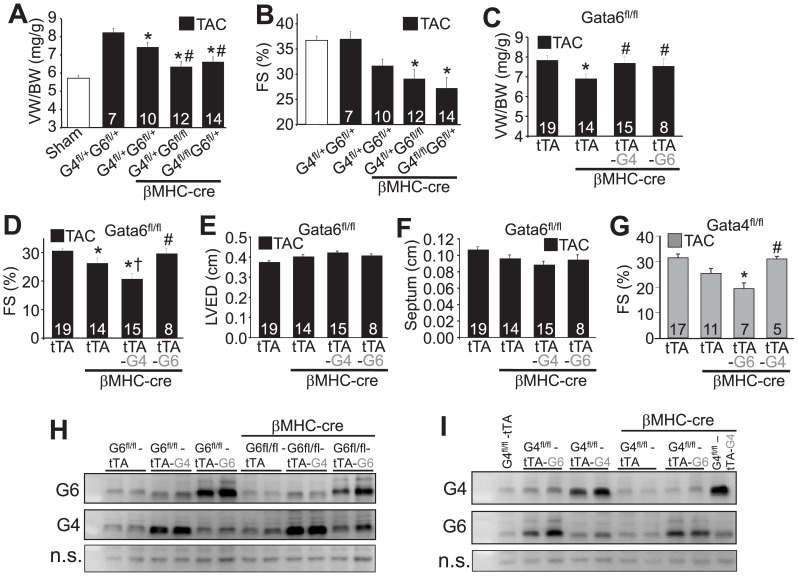
GATA-4 and GATA-6 play redundant roles in programming cardiac hypertrophy but not in adapting functional compensation. A, Ventricular weight to body weight (VW/BW) in the indicated genotypes of mice at 10–12 weeks of age following 2 weeks of TAC stimulation. The number of mice analyzed is shown in each bar of the graph. *P<0.05 versus *Gata4^fl/+^ Gata6^fl/+^*; #P<0.05 versus *Gata4^fl/+^ Gata6^fl/+^* with the βMHC-cre transgene. B, Fractional shortening (FS%) from the indicated mice at 10–12 weeks of age following sham or 2 weeks of TAC stimulation. The number of mice analyzed is shown in each bar of the graph. *P<0.05 versus *Gata4^fl/+^ Gata6^fl/+^* with the βMHC-cre transgene. C, VW/BW in the indicated groups of mice after 2 weeks of TAC stimulation at 10–12 weeks of age. The number of mice analyzed is shown in each bar of the graph. *P<0.05 versus *Gata6^fl/fl^* tTA transgene; #P<0.05 versus *Gata6^fl/fl-βMHC-cre^* tTA transgene. Abbreviations; tTA-G4, tetracycline transactivator transgene with the GATA-4 (G4) transgene; tTA-G6, tetracycline transactivator transgene with the GATA-6 (G6) transgene. D, Fractional shortening to measure ventricular performance of the same mice described in “C”. *P<0.05 versus *Gata6^fl/fl^* tTA transgene; ^†^P<0.05 versus *Gata6^fl/fl-βMHC-cre^* tTA transgene; #P<0.05 versus *Gata6^fl/fl-βMHC-cre^* tTA transgene or *Gata6^fl/fl-βMHC-cre^* tTA transgene with GATA-4 transgene. E, F, Echocardiography measured left ventricular end diastolic dimension (LVED) and septal thickness in hearts of the indicated groups of mice after 2 weeks of TAC. G, Fractional shortening in *Gata4* heart-specific, deleted mice with the additional transgenes shown after 2 weeks of TAC stimulation. Number of mice analyzed is shown in the bars. *P<0.05 versus *Gata4^fl/fl^* tTA transgene; #P<0.05 versus *Gata4^fl/fl-βMHC-cre^* tTA transgene with the GATA-6 transgene. H, I, Western blot analysis for GATA-4 (G4) or GATA-6 (G6) protein from the hearts of the indicated mice. A non-specific (n.s.) band from each of the western blots was used to show equal loading and running conditions.

To more definitively examine if GATA-4 and GATA-6 function in a dosage-dependent, redundant manner in the adult heart we also analyzed mice with cardiac-specific deletion of multiple *Gata4* and *Gata6* alleles. Here we analyzed *Gata4-loxP* (fl) and *Gata6-loxP* targeted mice crossed with mice expressing the cre recombinase cDNA from the βMHC promoter, which we have previously shown results in highly efficient deletion of each gene in the heart resulting in a reduction in the pressure overload-induced hypertrophic response [4], [6]. For the current analysis we compared double heterozygous mice of *Gata4^fl/+^ Gata6^fl/+^* versus three allele deleted mice consisting of *Gata4^fl/fl^ Gata6^fl/+^* or *Gata4^fl/+^ Gata6^fl/fl^* following 2 weeks of transverse aortic constriction (TAC) to induce hypertrophy (Fig. 2A). Control mice were double heterozygous without the βMHC-cre transgene, which were identical to wildtype in their response (Fig. 2A). The data show a significant attenuation of the hypertrophic response in double heterozygous targeted mice, which was significantly reduced again by further deletion of 3 out of 4 *Gata4/6* alleles in either combination (Fig. 2A). Similarly, analysis of cardiac function by echocardiography after TAC stimulation also showed a similar dosage-dependent reduction corresponding with the number of *Gata4/6* alleles deleted (Fig. 2B). We have previously reported that 4/4 allele targeted mice crossed with mice containing the βMHC-cre transgene resulted in spontaneous heart failure at baseline and complete lethality by 16 weeks of age, hence we were not able to perform TAC stimulation on fully homozygous *Gata4/6* loxP-targeted mice [6]. Taken together, our results suggest that GATA-4 and GATA-6 function equivalently and in a dosage-dependent manner in programming the hypertrophic response.

We also employed an entirely independent approach in which we bred mice containing either the GATA-4 or GATA-6 heart-specific transgene into the *Gata4* and *Gata6* heart-specific gene-deleted genetic backgrounds. This replacement transgenesis approach restored the full hypertrophic potential of the heart after TAC, further suggesting that these 2 transcription factors function redundantly in programming the cardiac growth response (Fig. 2C). However, only the GATA-6 transgene restored the ability of *Gata6* cardiac-specific deleted mice to compensate and resist heart failure after TAC stimulation, as the GATA-4 transgene replacement in the *Gata6* null background produced even slightly worse cardiac function after TAC (Fig. 2D), although left ventricular end diastolic dimension and septal thicknesses were not significantly changed (Fig. 2E and F). By comparison, analysis of the reciprocal cross in *Gata4* heart-specific, deleted mice showed that only the GATA-4 transgene prevented functional decompensation, while the GATA-6 transgene was ineffective (Fig. 2G). Western blotting from all these crosses showed transgene generated GATA-4 or GATA-6 protein expression in each of the genetic backgrounds analyzed here (Fig. 2H and I). As an aside, if one examines these western blots carefully the deletion of *Gata4* or *Gata6* protein with the βMHC-cre transgene does not appear obvious, although we have previously shown that this strategy results in 70–90% loss of each protein [4], [6]. The differences here are likely do to the timing of these experiments, which were from older animals that accumulate other non-myocytes in the heart that also express GATA-4 and GATA-6, as well as from mild cross reactivity of the GATA-4 and GATA-6 antibodies used here. Indeed, a 50% reduction in GATA-6 protein is observable in *Gata6^fl/fl^* mice with the βMHC-cre transgene versus *Gata6^fl/fl^* mice without the cre transgene (Fig. 2H, lanes 1+2 versus 7+8). However, lanes 9+10 show higher levels of GATA-6 protein because of GATA-4 overexpression in these mice and the fact that the GATA6 antibody partially cross-reacts with GATA4. The same general phenomena are present in figure 2I. In conclusion, while GATA-4 and GATA-6 function in a dosage-dependent and redundant manner in programming the cardiac growth response, each must have unique transcriptional targets to explain why reciprocal replacement does not prevent decompensation with 2 weeks of pressure overload stimulation. Only 2 weeks of pressure overload stimulation was performed because decompensation was already apparent at this time, hence longer time periods of simulation were not necessary and lethality becomes more of a confounding factor as timing is extended.

**Figure 3:**
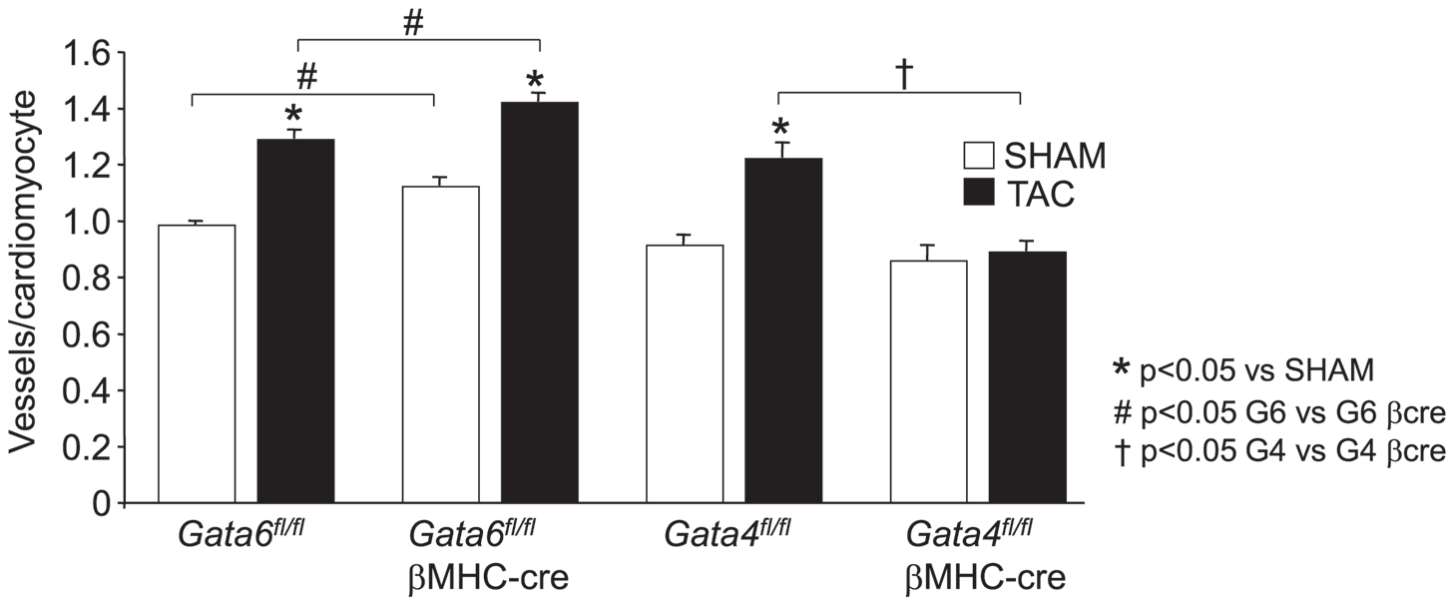
Cardiac-specific deletion of *Gata4* but not *Gata6* prevents compensatory angiogenesis in the hearts of mice. The graph shows immunohistological quantitation of capillaries in the left ventricle normalized to surrounding cardiomyocytes in the indicated groups of mice. TAC was performed for 2 weeks. Mice were 10–12 weeks of age at harvesting. Hearts from at least 4 mice were analyzed in each group. *P<0.05 versus *Gata6^fl/fl^* or *Gata4^fl/fl^* sham.

To further investigate how GATA-4 and GATA-6 might function differently in the heart we performed extensive gene array analysis for altered cardiac mRNA expression between *Gata4* and *Gata6* heart-specific deleted mice at 2 months of age (Table 1). A number of genes were identified as significantly changed in hearts between *Gata6^fl/fl-^* ^βMHC-cre^ versus *Gata4^fl/fl-^* ^βMHC-cre^ mice. The most significantly altered genes or ones with interesting regulatory connotations were selected for qRT-PCR confirmation (Table 2). Some of the more prominently differentially expressed genes were *Drd2*, *Gm129*, *Hey2*, *Bhlhb9*, *Adh1*, and *Irx5*. There were also interesting angiogenesis-related genes that were altered, hence qRT-PCR was run against a larger panel of such genes to extend and confirm the array results, similar to a previous description in the literature (Table 3, ref 12). For example, *Gata4*-deleted hearts showed a downregulation in *Vegf1a* and *Fgf16*, which were not changed in *Gata6*-deleted hearts. Indeed, we have previously shown that hearts from *Gata4* cardiac-specific targeted mice had a defect in cardiac angiogenesis following stress stimulation that then directly predisposed to heart failure, although the role of *Gata6* in this process was not analyzed [5]. To further assess this one potential mechanism, we performed TAC stimulation in both *Gata4* and *Gata6* heart-specific gene-deleted mice and then assessed capillary density in the heart (Fig. 3). The data show that wildtype controls (*Gata6^fl/fl^* mice) have the known characteristic increase in capillary density after TAC stimulation, while the *Gata4* heart-specific deleted mice are defective in this response (Fig. 3). We have previously shown that *Gata6^fl/fl^* mice are entirely the same as wildtype mice and that the Gata6 locus is otherwise unaffected by the placement of the loxP sites [6]. Unlike *Gata4* heart-specific, deleted mice, *Gata6* deleted mice showed no defect in their ability to augment angiogenesis after TAC stimulation (Fig. 3). These results suggest at least one prominent mechanism through angiogenesis whereby the transcription factors GATA-4 and GATA-6 can function differently in the heart, thus having a differential effect in predisposing to heart failure with stress stimulation.

**Table 1:** Differentially regulated genes from microarray.

Gene symbol	Description	ΔGATA-6	ΔGATA-4	Difference
Timp4	Tissue inhibitor of metalloproteinase 4	−1.18	3.00	3.56
Angptl7	Angiopoietin-like 7	1.05	2.39	2.28
Spink4	serine peptidase inhibitor, Kazal type 4	1.02	2.11	2.07
Gcnt1	Glucosaminyl (N-acetyl) transferase 1	−1.17	2.89	2.03
Ptgfr	Prostaglandin F receptor	−1.00	2.92	1.92
Avpr1a	Arginine vasopressin receptor 1A	1.06	1.58	1.49
Zbtb16	Zinc finger and BTB domaincontaining 16	−1.17	2.10	1.24
Bhlhb9	Basic helix loop helix domain containing class B9	−1.05	2.18	1.23
Scgb1c1	Secretoglobin 1C1	−1.01	2.09	1.10
Gm129	Gene model 129	−1.10	2.00	1.09
Fmo2	Flavin containing monooxygenase 2	−1.10	1.83	0.92
Gsta3	Glutathione S transferase, alpha 3	−1.04	1.81	0.85
Adh1	Alcohol dehydrogenase 1	−1.28	1.61	0.83
Rhobtb1	Rho related BTB domain containing 1	−1.09	1.61	0.69
Tsc22d3	TSC22 domain family 3	−1.05	1.64	0.69
Wee1	Wee 1 homolog	−1.06	1.57	0.63
Pfn2	Profilin 2	−1.02	1.61	0.63
Bc055107	Family with sequence similarity 107, A	−1.13	1.51	0.63
Fstl4	Follistatin like 4	−1.37	1.34	0.61
Fkbp5	FK506 binding protein 5	−1.20	1.40	0.57
Gadd45g	Growth arrest and DNA damageinducible 45γ	−1.06	1.53	0.56
Bcl2l1	BCL2 like 1	−1.08	1.45	0.52
Ifi205	Interferon activated gene 205	−1.15	1.38	0.51
Tchhl1	Trichohyalin like 1	−1.02	1.45	0.47
Thrsp	Thyroid hormone responsiveSPOT14 homolog	−1.46	1.14	0.46
Il33	Interleukin 33	−1.03	1.42	0.45
Trdn	Triadin	−1.26	1.25	0.45
Ankrd10	Ankyrin repeat domain 10	−1.02	1.42	0.44
Ddit4	DNA damage inducible transcript 4	−1.02	1.42	0.44
Tom1l1	Target of myb1 like 1	−1.06	1.38	0.44
Cebpb	CCAAT/enhancer binding protein(C/EBP) beta	−1.08	1.35	0.42
Efna5	Ephrin A5	−1.04	1.38	0.42
Rgs2	Regulator of G-protein signaling 2	−1.01	1.41	0.42
Hey2	Hairy/enhancer of split related withYRPW motif 2	−1.35	1.09	0.35
Drd2	Dopamine receptor 2	1.57	−1.18	0.73
Nppb	Natriuretic peptide precursor type B	1.55	−1.04	0.59
Egr1	Early growth response 1	1.19	−1.62	0.58
Fbn1	Fibrillin 1	1.26	−1.41	0.55
Eg633640	Predicted gene	1.31	−1.30	0.53
Rrad	Ras-related associated with diabetes	1.00	−2.09	0.52
Ptk2b	PTK2 protein tyrosine kinase 2 beta	1.26	−1.35	0.51
Trmt5	TRM5 tRNA methyltransferase 5homolog	1.11	−1.65	0.50
Syk	Spleen tyrosine kinase	1.19	−1.43	0.49
Irx5	Iroquois related homeobox 5	1.26	−1.29	0.48
Emr1	EGF-like modulecontaining, mucin-like	1.14	−1.42	0.43
Kcnip2	Kv channel interacting protein 2	1.11	−1.44	0.42
Loxl1	Lysyl oxidase like 1	1.18	−1.26	0.38
Col15a1	Procollagen XV	1.17	−1.27	0.38
Ssync	Syncoilin	1.27	−1.12	0.38
Arhgef19	Rho guanine nucleotideexchange factor 19	1.24	−1.14	0.36
Ptprc	Protein tyrosine phosphatase,receptor type C	1.05	−1.44	0.36
Aurka	Aurora Kinase A	1.26	−1.10	0.35
Rasl11b	RAS-like family 11member B	1.03	−1.49	0.35

Adult heart mRNA was collected from *Gata6^fl/fl-βMHC-cre^* mice and compared against mRNA from *Gata6^fl/fl^* to generate Affymetrix expression arrays of genes that were specifically changed in with deletion of *Gata6*. And identical protocol was used to analyze genes changed with deletion of *Gata4* from the heart. Afterwards the two arrays sets were cross compared to examine genes that might be more specific to GATA-4 or GATA-6 regulation, which is represented as the column “difference” showing normalized expression results. The values represent the difference between normalized GATA-4 expression over normalized GATA-6 expression, both of which are corrected to the common cre control and each respective floxed line control. The negative numbers represent downregulated genes. Gene names are shown in the left column and protein names are shown in the very next column.

**Table 2:** qRT-PCR confirmation of differentially regulated genes.

Gene name	*Gata6fl/fl*	*Gata6fl/fl* βMHC-cre	*Gata4fl/fl*	*Gata4fl/fl* βMHC-cre
Gctn1	1	0.93±0.24	1	1.56±0.18
Ptgfr	1	2.78±0.43	1	2.76±0.34
Zbtb16	1	0.87±0.14	1	1.23±0.16
Bhlhb9	1	1.18±0.14	1	2.98±0.30
Scgb1c1	1	1.75±0.30	1	2.66±0.24
Gm129	1	1.86±0.45	1	0.98±0.20
Per1	1	1.40±0.32	1	0.78±0.11
Fmo2	1	0.99±0.15	1	2.01±0.20
Gsta3	1	1.55±0.21	1	3.15±0.27
Adh1	1	0.85±0.12	1	2.36±0.24
Rhobtb1	1	1.07±0.12	1	2.35±0.20
Tsc22d3	1	1.42±0.17	1	2.10±0.17
Wee1	1	1.29±0.21	1	2.02±0.15
Pfn2	1	2.09±0.29	1	2.98±0.25
Fkbp5	1	0.75±0.09	1	1.32±0.13
Gadd45g	1	1.43±0.20	1	1.38±0.12
Avpr1a	1	1.00±0.11	1	2.37±0.30
Bcl2l1	1	1.24±0.12	1	1.91±0.15
Trd	1	1.03±0.12	1	1.65±0.12
Ankrd10	1	1.60±0.18	1	3.09±0.24
Cebp	1	1.10±0.16	1	1.61±0.17
Efna5	1	1.65±0.19	1	2.02±0.15
Rgs2	1	1.57±0.24	1	1.40±0.16
Hey2	1	1.32±0.16	1	2.97±0.43
Drd2	1	4.20±0.57	1	0.47±0.06
Egr1	1	0.93±0.11	1	0.93±0.09
Zfp52	1	0.96±0.01	1	0.98±0.00
Fbn1	1	1.73±0.22	1	1.81±0.15
Gm7120	1	1.85±0.31	1	1.54±0.14
Rrad	1	1.38±0.16	1	1.35±0.11
Ptk2b	1	0.86±0.10	1	1.26±0.15
Irx5	1	2.73±0.40	1	1.37±0.17
Arhgef19	1	0.83±0.14	1	1.28±0.14
Ptprc	1	1.06±0.21	1	0.62±0.07
Aurka	1	1.21±0.13	1	1.91±0.21
Rasl11	1	1.86±0.22	1	1.17±0.12
Timp4	1	0.88±0.08	1	1.60±0.23
Fstl4	1	0.79±0.10	1	1.63±0.16

Adult heart mRNA was collected from the indicated lines of adult mice and subjected to qRT-PCR to analyze for differences in gene expression to verify or extend the Affymetrix results shown in Supplemental Table 1. Gene names are shown in the left column. Values are set relative to expression in *Gata6^fl/fl^* or *Gata4^fl/fl^* controls.

**Table 3:** qRT-PCR of differentially regulated angiogenesis and other genes.

Gene name	*Gata6 fl/fl*	*Gata6fl/fl* βMHC-Cre	*Gata4fl/fl*	*Gata4fl/fl* βMHC-Cre
Nppa	1	1.26±0.14	1	0.80±0.11
Nppb	1	1.41±0.24	1	2.33±0.25
Atp2a2	1	1.29±0.15	1	1.64±0.13
Gata4	1	1.87±0.19	1	0.25±0.03
Zfpm2	1	1.28±0.13	1	3.33±0.45
Ctf1	1	1.70±0.18	1	1.92±0.18
Kcne1	1	0.21±0.06	1	0.91±0.15
Foxc1	1	2.22±0.26	1	1.39±0.14
Vegf1a	1	0.98±0.10	1	0.57±0.07
Vegfb	1	1.69±0.20	1	1.52±0.13
Vegfc	1	1.52±0.15	1	1.64±0.14
Fgf1	1	1.06±0.19	1	1.43±0.19
Fgf2	1	1.43±0.19	1	1.77±0.20
Fgf9	1	1.46±0.16	1	1.78±0.22
Fgf12	1	4.77±0.60	1	1.01±0.10
Fgf16	1	1.74±0.29	1	0.59±0.08
Thbs	1	0.65±0.07	1	1.12±0.14
Timp1	1	1.75±0.19	1	2.35±0.38
Timp2	1	1.42±0.14	1	1.99±0.16
Hpse	1	1.33±0.13	1	1.85±0.19
Col15a1	1	2.24±0.26	1	1.43±0.11
Col18a1	1	1.36±0.15	1	1.33±0.13
Ctgf	1	3.96±0.57	1	1.77±0.19
Fn1	1	1.49±0.15	1	1.82±0.14

Adult heart mRNA was collected from the indicated lines of adult mice and subjected to qRT-PCR to analyze for differences in gene expression for the shown set of angiogenesis related genes in the left most column. Values are set relative to expression in *Gata6^fl/fl^* or *Gata4^fl/fl^* controls.

## Discussion

The existence of gene duplication in evolutionary biology poses many interesting questions, as members of related gene families can have a spectrum of similar versus more specialized functionality. Typically, the greater the evolutionary distance underlying a gene duplication event, the greater the functional divergence. GATA-4 and GATA-6 are nearly identical in their primary sequences within the DNA binding zinc-finger domains, with large areas of partial sequence conservation outside this motif [2]. GATA-4 and GATA-6 have been suggested to function differently with respect to binding select cardiac-specific promoters, as well as to synergize when co-transfected with the BNP promoter construct [10]. Such results suggested that these 2 factors had unique functions in the heart, especially as might relate to the cardiac hypertrophic response or adult heart gene expression. The very concept that these 2 factors likely function differently in the heart is in basic agreement with our results, as the cardiac GATA-4 transgene could not functionally compensate for the deletion of *Gata6* from the heart, and the cardiac GATA-6 transgene could not functionally compensate for the loss of *Gata4*, yet each did effectively compensate for itself suggesting that the approach was valid (i.e., GATA-6 transgene into the *Gata6* deleted background). Thus, these functional results suggested that there is a set of genes uniquely regulated by either GATA-4 or GATA6 in the heart, which we indeed confirmed by gene array analysis. The most prominent and mechanistically important of which appears to be select angiogenic genes as effectors of adaptation in the heart following pressure overload stimulation. However, with respect to the hypertrophic response *per se*, we observed that GATA-4 and GATA-6 were redundant and acted in concert as part of a dosage of total GATA protein necessary for efficient induction of growth regulating genes in the heart. Indeed, *Gata4* and *Gata6* genes were shown to be redundant in programming cardiomyogenesis during development [7], and *Gata4^+/−^ Gata6^+/−^* double heterozygous mice are embryonic lethal yet each single heterozygote is fully viable [8], again suggesting redundancy in function and that total gene dosage is the more important determinant for embryonic development. Hence, the *Gata4* and *Gata6* genes appear to be at an intermediate stage of evolutionary genetic drift where they have acquired unique functionality but also retain a degree of overlap, such as in regulating the adult hypertrophic response. Overexpression of GATA-4 or GATA-6 in the heart, as these mice age out past 6 months, each appears to program a similar extent of maladaptive cardiac hypertrophy, while cardiac-specific deletion of either *Gata4* or *Gata6* from the heart appears to render the heart less adapted and more prone to failure, hence both genes appear to also be necessary for optimal cardiac “health” and physiology [4], [6], [11].
